# Integrating basic sciences into clerkship rotation utilizing Kern’s six-step model of instructional design: lessons learned

**DOI:** 10.1186/s12909-024-05030-z

**Published:** 2024-01-17

**Authors:** Satwat Hashmi, Qamar Riaz, Husnain Qaiser, Saira Bukhari

**Affiliations:** 1 Department of Biological and Biomedical Sciences, Faculty of Health Sciences, Aga Khan University, Stadium Road, 74800 Karachi, P.O. Box 3500, Pakistan; 2 Department of Educational Development, Department of Surgery, Faculty of Health Sciences, Aga Khan University, Karachi, Pakistan; 3 Department of Medicine, Section of Cardiology, Faculty of Health Sciences, Aga Khan University, Karachi, Pakistan

**Keywords:** Integration, Basic sciences, Medical clerkship, Kern’s instructional design, Medical education

## Abstract

**Background:**

It is generally agreed that basic and clinical sciences should be integrated throughout the undergraduate medical education, however, there is still need for continued formal integration of basic sciences into clinical clerkship in many medical schools across the globe.

**Methods:**

Utilizing Kern’s Six-Step Model of Instructional Design, we aimed to develop an intervention that would facilitate cognitive integration of basic and clinical sciences. After problem identification and targeted needs assessment through focused group discussion with the students and faculty, objectives were devised with an implementation plan of using flipped class approach to develop a content-focused and learner-centered teaching strategy. This intervention was piloted in the 2-week cardiology clerkship in Year 5. Evaluation of the content, integration, student and faculty experiences were recorded through in-depth interviews, FGDs and a formative MCQ test.

**Results:**

Flipped classroom based integrated sessions were successfully developed. The implementation phase was met with challenges that primarily stemmed from the diverse teaching styles among faculty members, hesitance to deviate from conventional practices, variations in clinic timings, and demanding schedules. Noteworthy observations were in terms of ownership of the project, the need for faculty development in modern student-centered teaching pedagogies, opportunities for content improvement, scheduling of sessions, and suggestion of revisiting fundamental concepts in basic sciences through a brief boot camp-style session at the onset of the clerkship. The role of flipped case model and clinical cases in integrating basic sciences into clinical sciences were appreciated by the students. Standardization in teaching practices was identified as the major challenge by the faculty.

**Conclusions:**

A functional, learner-centered framework of cognitive integration of basic sciences in clinical sciences curriculum of cardiology rotation was developed with a potential to be implemented in other clerkship rotations.

**Supplementary Information:**

The online version contains supplementary material available at 10.1186/s12909-024-05030-z.

## Background

Deep understanding of basic science concepts plays a crucial role for effective clinical decision making among doctors [1–3]. It enhances the ability to relate new learning to past information and problem solving [4] and hence to excellence in clinical performance [5].

There is general consensus that basic and clinical sciences throughout the undergraduate medical education should be integrated [6]. Integration refers to abandoning the traditional discipline based discreet segmentation of teaching and learning activities. Literature is rife with evidence that if integration is targeted towards developing cognitive connections between the basic and clinical domains; learning becomes effective. Integration promotes transfer of knowledge effectively [7] and improves diagnostic skills in early learners [8]. Studies have shown that students trained in an integrated curriculum outperformed students trained in the traditional curriculum in accurate diagnosis of clinical presentation [9]. Many medical schools are now using different strategies for integration throughout the medical curriculum [10, 11] to develop a conceptual, cognitive connection between basic and clinical domains for the students to become reflective medical practitioners [2, 12].The ‘integration ladder’ proposed by Ronald Harden [13], defines the steps between the two extremes of subject based and integrated teaching. This guide is very useful to evaluate the level of integration in different institutions.

At our institution, integration of basic and clinical sciences is provided by PBLs (Problem based learning) and PSILs (Problem solving integrated learning) in addition to a bench -to-bedside module in Year 3. This model is successful and is a good starting point but there is a need to move further up the ladder with a conscious attempt to create cognitive integration.

As students’ progress through clinical years, they are more focused on clinical sciences and lose touch with basic sciences. Basic science is the cornerstone of understanding complex clinical scenarios and the development of new disease pathophysiology and management models. It is important to remain keyed in with basic sciences at a time when clinical science forms the dominant part of curriculum i.e., Year 5 of medical school.

Currently, there is a lack of continued formal integration of basic sciences into clinical clerkship in our as well as other medical schools across the globe [14–16].

Utilizing Kern’s Six-Step Model of Instructional Design, we aimed to develop an intervention that would facilitate cognitive integration of basic and clinical sciences. To achieve this goal, content-focused, session-oriented and learner-centered strategies were adopted [12].

We decided to pilot an intervention in the cardiology clerkship, which is one of the smaller clerkships in Year 5. The cardiology clerkship is a 2-week rotation that is part of the larger Internal medicine rotation at our institution, the Aga Khan University.

We expected that enriching the learning experience of Year 5 students in cardiology clerkship rotation will deepen their basic science knowledge within a meaningful clinical context and will also help us in gathering evidence for curriculum modification of other clerkship rotations at our institution.

## Methods

Kern’s model of instructional design [17] include the 6 steps; (1) General Needs Assessment.

(Problem Identification); (2) Targeted Needs Assessment; (3) Goals and Objectives; (4) Educational Methods and Strategies; (5) Implementation; and (6) Evaluation. We chose the cardiology clerkship rotation for year 5 medical students to design, develop and implement this educational intervention. There are a total of 4 rotations in Year 5 including surgery, medicine, family medicine-emergency medicine and electives. The 2-week cardiology rotation is a sub-rotation of the Medicine clerkship during which 5–7 students rotate.

The project had the approval of the institutional Ethics Review Committee (ERC-2021-6729).

## Results

### STEP-1: Problem identification and general needs assessment

The need for fortifying the basic science concepts in clinical clerkship years was being felt through general feedback of clinical teachers and students alike. There was constant discussion in curriculum meetings and in classrooms about the need for the students to form a critical connection between the clinical condition and the basic science behind it. Anecdotal evidence from Year 5 students also suggested the need for such an intervention citing difficulty in recalling the basic science facts when tested in the Final Year professional exam. To tackle this issue a team got together consisting of a basic scientist, a medical educationist, and a practicing physician. The team members were part of the various medical college educational committees and were closely involved in the development and delivery of medical curriculum to preclinical and clinical years. The team began its work by first doing a thorough and extensive literature review to investigate various strategies already in use for integrating basic sciences in clinical rotations which satisfies the basic principals and aim of integration and would be a good fit for our system. The committee concluded that there was not a single best recommended way of integrating basic sciences concepts and the team had to come up with an innovative solution to design, develop and implement educational strategies that would be practical and a feasible fit in our system/ institute.

### STEP-2: Targeted needs assessment

In the second step of Kern’s approach of targeted needs assessment, we conducted two focused group discussions (FGD), one with 6 Year 5 undergraduate medical students and the other with 7 cardiology clinical faculty including cardiology clerkship coordinator. The participants were recruited by purposive sampling technique for both the faculty and medical students.

The FGD interview guide was developed based on relevant literature with reference to the issues identified during our general needs assessment process (Appendix-1). The FGD sessions were facilitated by a faculty trained in qualitative research. The FGDs were 50–60 min long and were audio taped for verbatim transcription. Content analysis of the FGD was performed independently by two authors and agreement on the emerging themes was reached after discussion.

The following main themes emerged from both the FGDs:

1. Sub-optimum/inadequate clinical and basic science integration: The integration at both the pre-clinical and clinical years was suboptimal during the clerkship. Students said they failed to see the clinical application of basic sciences knowledge until the final year.
2. Use of traditional/ didactic teaching methods: While the curriculum is delivered through traditional lectures and PBLs in addition to newer teaching modalities like TBLs during the preclinical years, the students and faculty both were not convinced of the utility of the educational methods in successfully linking the basic sciences with the clinical sciences. The clinical departments have academic schedules comprising of tutorials and student presentations, with focus only on the clinical aspects of the diseases with little or no opportunity to discuss or revise basic science concepts.
3. Time constraints & stakeholder expectations: Both faculty and students had contrasting expectations during clerkships. The faculty expected that the students should have thorough knowledge of the basic science principles before they start their clerkship so they can build on the clinical knowledge over it. Students on the other hand said that they had usually forgotten their basic sciences by the time they started their clerkships and therefore expected that the relevant basic science content should be revised at the beginning of such rotations. However, both faculty and students identified that revising basic sciences at the beginning of each rotation was not feasible due to time constraints.

As intended, the targeted needs assessment provided us the detailed information about the content being taught, the strategies being currently used to deliver this content, and the gaps in the current instructional strategies that need to be addressed. We were in an excellent position to develop focused goals and objectives for new educational intervention for each session identified through the discussions.

### STEP-3: Goals and objectives

In the third step of Kern’s approach, goals and objectives for the intended course were developed as follows:

By the end of the rotation, the students will be able to:

1. Relate the basic science concepts with the clinical presentations of common cardiac pathologies seen during cardiology clerkship.
2. Revisit the basic science concepts in ordering and interpreting laboratory, electronic and radiologic investigations pertinent to cardiac conditions.
3. Apply basic science principles in developing therapeutic and non-therapeutic management plans for the common cardiac pathologies.

### STEP-4: Educational methods and strategies

After thorough review of the curriculum for the cardiology clerkship and points discussed in the FGDs, we identified 4 lecture-based sessions in the two-week cardiology rotation that had a focus on disease processes that are anchored in basic science and are reinforced by patient care experience.

We decided to use the existing opportunities for didactic teaching and learning so that time for their clinical exposure is not taken up. We were mindful that faculty and the students are not overburdened. The educational intervention for the intended objectives was developed following the flipped class model.

The relevant reading resources were identified and shared with the students two days before starting the rotation. We prepared these pre readings handouts from different sources including research articles, book chapters and free internet educational resources. The research articles used in developing the content for handouts were referenced and the students were encouraged to read them as an additional resource. The already existing lecture sessions were converted to active learning sessions by using case-based learning and discussions. This was also supplemented by brief interactive lectures to reinforce or clarify essential concepts while integrating basic and clinical sciences. A few formative quizzes were also introduced to enhance learning and retention of knowledge. A sample session had the following components:

- Pre reading (basic science concepts) comprising of handouts developed from book chapters and free internet resources shared through VLE (virtual learning environment).
- In-class session: clinical case discussion to link already learned basic science concepts with the clinical signs and symptoms.
- Mini lecture on clinical management prepared by the clinical faculty.
- Quiz for formative assessment.

A separate formative assessment comprising of 50 cardiology MCQ questions was developed following the principles of item development to assess their integrated basic and clinical science knowledge. It was scheduled at the end of the academic year.

### STEP-5: Implementation

The educational intervention was implemented starting with the first cardiology sub-rotation of the academic year and continued throughout the year. An orientation session was conducted with the faculty where the purpose of the educational intervention and the format of the intervention was discussed.

The educational material for pre-reading and clinical cases was shared with the faculty during orientation session and detailed discussion regarding the session format i.e., the case-based discussions, mini lectures and formative assessments were conducted to ensure similar learning experience within all session by all students throughout all the rotations.

All the students were sent emails at the start of the rotation with the link to the relevant pre-reading material on the Institutional Virtual Learning Environment (VLE). The 4 lecture-based sessions were conducted over a 2-week rotation time. The faculty incorporated various educational strategies mentioned in step 4 to integrate basic science concepts into case base discussions. Throughout the year, the faculty was reminded to take the integrated sessions and conduct informal quizzes during sessions to assess and promote student learning.

At the end of the academic year, the formative 50-MCQ test was administered. An email was sent to the students regarding the time and date of the test. The test was conducted online via VLE and the duration to complete it was 60 min. The responses were recorded, and the data was gathered in the end. This same formative test had also been conducted for the previous cohort class of 2021 who did not undergo the educational intervention at the end of their academic year to assess the effect of the intervention.

### STEP-6: Evaluation and feedback

This phase focused on three main areas:

- Evaluation of the content, integration and faculty experience. This was recorded by an in-depth-interview (Appendix-2) at the end of the project with 2 faculty members who had been involved in the implementation of the intervention.
- Evaluation and feedback of the medical student experience of this educational intervention was evaluated by another FGD with a group of 6 students (Appendix-2).
- Student’s performance was assessed by comparing the scores of the current cohort with that of the earlier cohorts in the VLE based MCQ exam offered to the students at the end of Year 5 with questions made specifically to test basic science links to clinical cardiology concepts.

### In depth interview

In depth interviews were conducted with two faculty members who were consultant cardiologists and participated in the implementation of the flipped classroom sessions.

Both the members felt very strongly about the need for and utility of these integrated sessions. “*I feel that basic sciences are extremely important for connections. We underplay it, we are not as good as we could have been if we had simply found time or felt an interest towards revising and applying basic science concepts to clinical science”*. One of the faculty members explained how she managed the flipped sessions; *“My session had 15 minutes for revision, 30 minutes for case scenarios and these would be variable. Then the last 15 minutes were for a quiz. This way my 1 hour used to go very smoothly”*. The other faculty member gave a practical example. *“I diurize a patient and the creatinine improved, so shouldn’t diuresis increase the creatinine? Why does the creatinine decrease, then they (the students) thought about it and a few of them came up with the idea that Frank-Starling curve improved for the patient because the preload was decreased. So, this was a kind of implementation that they enjoyed rather than talking about Frank-Starling in detail”.*

Both faculty members felt that the intervention partially achieved its intended outcome. For some students the sessions worked very well but for others it did not turn out to be very productive. The main reason cited by both the faculty members was that the students did not come prepared for the sessions. A lot of students had not seen the pre reading material that was sent to them through email. There was a communication problem with a few groups. *“Students did not realize that the pre-reading material was there and was essential for the session to work. They did not know why it was required”*. Some of the faculty members tried to mitigate this problem by going over the content again in the class, *“So I tried to compensate for that by teaching them what they were supposed to know before coming to the class so that actually took up a lot of time. So, that was one of the problems with the intervention”*. The other reason that led to sub optimum results of the intervention was that some of the faculty members did not implement the sessions according to the structure provided in the intervention plan. *“When it came to practice it was not done as it should have been since some people simply did not do it and some people did it partially and some people did it the way it was meant to be. There was a lot of variety, but I feel the idea suffered from inertia because most faculty does not feel very comfortable to change their practices and implement something new because they have busy schedules, and they just have that one hour to get the message across”*. Both our interviewees agreed that standardization of these sessions was a key issue.

When asked about the recommendations for future implementation, one of our interviewees suggested giving short videos for pre-session preparation instead of written material. The other faculty thinks that 1–2 pages of simplified pre reading would be easier for the students to follow. It was also suggested that if there was a template given to all faculty members that they could use to organize their sessions, it would remind faculty members to adhere to the structure of the flipped session and will avoid discrepancy between faculty sessions, *“So perhaps we could give them a couple of slides, maybe 3–4 slides to use at the beginning of their presentation which would be distilled basic science concepts so that whatever presentation they use for their session, the 3–4 slides in the beginning are all standardized”*. Both the interviewees suggested to run another iteration of these sessions with incorporation of the feedback received from students and faculty as the importance of integrating basic with clinical sciences could not be overemphasized, *“I think integration is important. This doesn’t mean that you have to dedicate a lot of time. Touch the basic concepts in an organized way, and sessions are the best time for this. You should revise in a way that it builds the base for the upcoming 30 minutes”.*

### Focus Group Discussion

Students appreciated the overall effort, especially the flipped class model. Students greatly valued the efforts of the facilitators in linking the basic sciences with the clinical sciences in interpreting the investigations, especially the ECG and blood markers. Students also appreciated the use of clinical cases during sessions for better understanding of the underlying pathophysiology. However, they identified some areas that needed consideration during implementation. The students commented that not all the faculty used the recommended strategies in their sessions. They also shared that few of the reading materials were very good in summarizing and linking the basic and clinical sciences but not all. While discussing the reading material, most of the students preferred the handouts shared as reading materials because they were precise and concise, and thought that research articles were not very useful at their level and for this purpose. An important observation made by the students was that since these interventions were made using the already present framework of academic sessions, few topics that should have been learned at the beginning of the rotation were taught towards at the end of the rotation which left very limited time to apply that in clinics while interacting with the patients. They suggested having a 2-day boot camp like strategy either before starting the clerkship or the first two days of the clerkship when students can revisit all the relevant basic science while also relating it to the clinical science. When asked about their experience regarding the end of year formative MCQ test, 2 students who had undertaken the test said that it was very useful and helped them prepare for the final summative written examination. On asking why the other students didn’t take the test, they said that this specific test was scheduled close to their final written examinations, and thus was considered as a distraction because it only focused on one topic i.e., cardiology while exam had many other topics or components. One interesting finding was that those students who were preparing for USMLE exams or had already taken it, had very good understanding of basic science concepts, and were very confident in applying these concepts in clinical decision making.

### MCQ exam

The class of 2022 scored a mean of 62% (*n* = 15) in the MCQ exam at the end of year as compared to the class of 2021 who scored a mean of 52% (*n* = 17).

## Discussion

The concept of integration is not new to medical education [18]. Despite decades of effort, practical implementation of integration remains a challenge [10, 19]. Horizontal and vertical integration creates spaces within the curriculum for integrated teaching and learning to occur by creating proximity between two knowledge domains, however it remains unclear whether the logistic arrangement led to active integration for the student. Cognitive integration interweaves relevant basic science and clinical knowledge when reasoning through a patient problem [20]. Literature shows that purposeful teaching of foundational science, within a clinical context, promotes retention of knowledge [21].

Clinical problems encountered by physicians in their daily practice are complex and do not have simple solutions. Conceptual understanding of basic science principles with their clinical application is one way to train effective physicians. The organization and structure of the academic program thus needs to facilitate its function. This project was an attempt to bring this concept into focus and help set institutional priorities in this direction.

This pilot project was also an attempt to address an important gap in the process of curricular integration benefiting teaching practices. By trying it in a short clerkship, we wanted to test if we could create a model that could be followed in the other clerkship as well. By starting with a flipped classroom style sessions for the didactic sessions in the rotation we attempted to test whether the clinical faculty are invested in bringing this small change in their usual teaching practice that could benefit the students by making these sessions more uniform, standardized and in line with the concept of cognitive integration of basic and clinical sciences. We also hoped that it would encourage self-reflection on teaching practices across basic and clinical disciplines, which will pave the way for faculty development in this area. Enriching the learning environment through this cognitive integration of basic and clinical science discipline will further satisfy the tenet first coined by Knowles et al. [22] that adults learn best when the relevance of information is reinforced by its immediate application.

Our results show that despite the overwhelming support we received from the clinical faculty for the importance of this project and the value it would bring to the student learning experience through the targeted needs assessment, the faculty who actually tried to implement this project in its true spirit were few. The reasons ranged from variable teaching styles of different faculty, reluctance to deviate from standard practice, variable clinical timings, and extremely busy schedule.

The students also fully endorsed the usefulness of these sessions, but the reason cited by the students for the lukewarm response from the students was communication issues. The students felt that not enough emphasis was given by the faculty to ensure that students went through the pre reading material. The students felt that variability in the faculty’s preferred teaching style and in use of interactive methods to engage learners and ensure active learning were the main reasons that this intervention could not fully achieve its intended outcome.

Some very pertinent points have been identified. One of these is the ownership from the concerned faculty. Without their support, any intervention is likely to fail or remain under-implemented as happened in our study. Another point is about the importance of faculty development in student centered pedagogies. This is also evident from the literature that faculty beliefs and skills play an important role in implementing educational innovations at any level of education and training.

One very valid recommendation was the idea of using a 1–2 day boot camp before any clinical rotation that would focus only on revisiting basic science concepts so that the clerkship time could be used optimally for clinical learning. Different strategies could be used for teaching and learning during that boot camp, such as mini lectures, self-study reading material, videos or animations, or peer learning pairs/ groups.

Our study had some limitations. The duration of the clerkship rotation was short. The implementation and its effects might have been different in the case of longer clerkships. The other limitation was its soft implementation. As it was a pilot, neither the faculty nor the students felt compelled to use a certain strategy or resource for teaching or learning.

## Conclusion

In conclusion, this pilot project helped develop a functional, learner-centered framework of cognitive integration of basic sciences in the clinical sciences curriculum of Year 5 Cardiology which has the potential to be implemented as a regular feature in this rotation after incorporating valuable feedback received from faculty and students. This has also helped us gather evidence for what worked and what did not and will be instrumental in looking into the curricular reforms in other clerkship rotations.

### Electronic supplementary material

Below is the link to the electronic supplementary material.

Supplementary Material 1

Supplementary Material 2

## Data Availability

All data generated or analyzed during this study are included in this published article.
