# Supplementary material for: Integrating basic sciences into clerkship rotation utilizing Kern’s six-step model of instructional design: lessons learned
Source: BMC Med Educ. 2024 Jan 17;24:68. doi: 10.1186/s12909-024-05030-z (PMC10795218; doi:10.1186/s12909-024-05030-z)
Supplement: Supplementary file 1 — Supplementary Material 1 [file 12909_2024_5030_MOESM1_ESM.docx]

**Interview Questions for EVALUATION for FACULTY**

1. What is your opinion about the integration in the cardiology rotation- Explain?
2. Do you think it achieved the intended outcomes. Why?
3. What challenges did you face in the implementation?
4. What suggestion will you give to make it more effective?

**FGD guide for EVALUATION for STUDENTS**

1. What is your opinion about the integration in the cardiology rotation- Explain?
2. Do you think it achieved the intended outcomes. Why?
3. Did you face any challenges?
4. What suggestion will you give to make it more effective?
