# Supplementary material for: Integrating basic sciences into clerkship rotation utilizing Kern’s six-step model of instructional design: lessons learned
Source: BMC Med Educ. 2024 Jan 17;24:68. doi: 10.1186/s12909-024-05030-z (PMC10795218; doi:10.1186/s12909-024-05030-z)
Supplement: Supplementary file 2 — Supplementary Material 2 [file 12909_2024_5030_MOESM2_ESM.docx]

**Targeted Needs Assessment**

**FOCUSED GROUP DISCUSSION QUESTIONS FOR FACULTY**

1.        Do you think cardiology basic science concepts are essential for deeper understanding in order to solve the common cardiology related clinical problems?

2.        What is the current status of the student’s basic sciences knowledge when they come to cardiology rotation?

3.        What do you expect from a final year student coming to cardiology rotation regarding basic sciences foundational knowledge required?

4.        Where do you think is the problem?

5.        How do you suggest this could be addressed?

6.        List five basic sciences concepts/ topics that you think are essential for students to know in order to improve clinical understanding of common cardiology problems.

**FOCUSED GROUP DISCUSSION QUESTIONS FOR STUDENTS**

1.       How much basic science concept do you retain when you start your final year rotations?

2.       Do you think cardiology basic science concept are essential in deeply understanding and solving common clinical problem?

3.       If yes, how & when do you suggest this integration of basic & clinical should take place?
